# Structural Heterogeneities of the Ribosome: New Frontiers and Opportunities for Cryo-EM

**DOI:** 10.3390/molecules25184262

**Published:** 2020-09-17

**Authors:** Frédéric Poitevin, Artem Kushner, Xinpei Li, Khanh Dao Duc

**Affiliations:** 1 Department of LCLS Data Analytics, Linac Coherent Light Source, SLAC National Accelerator Laboratory, Menlo Park, CA 94025, USA; frederic.poitevin@stanford.edu; 2 Department of Mathematics, University of British Columbia, Vancouver, BC V6T 1Z4, Canada; rtkushner@alumni.ubc.ca (A.K.); xinpei.li@alumni.ubc.ca (X.L.); 3 Department of Computer Science, University of British Columbia, Vancouver, BC V6T 1Z4, Canada; 4 Department of Zoology, University of British Columbia, Vancouver, BC V6T 1Z4, Canada

**Keywords:** ribosome, cryo-EM, ribosome heterogeneity, ribosome evolution, conformational heterogeneity

## Abstract

The extent of ribosomal heterogeneity has caught increasing interest over the past few years, as recent studies have highlighted the presence of structural variations of the ribosome. More precisely, the heterogeneity of the ribosome covers multiple scales, including the dynamical aspects of ribosomal motion at the single particle level, specialization at the cellular and subcellular scale, or evolutionary differences across species. Upon solving the ribosome atomic structure at medium to high resolution, cryogenic electron microscopy (cryo-EM) has enabled investigating all these forms of heterogeneity. In this review, we present some recent advances in quantifying ribosome heterogeneity, with a focus on the conformational and evolutionary variations of the ribosome and their functional implications. These efforts highlight the need for new computational methods and comparative tools, to comprehensively model the continuous conformational transition pathways of the ribosome, as well as its evolution. While developing these methods presents some important challenges, it also provides an opportunity to extend our interpretation and usage of cryo-EM data, which would more generally benefit the study of molecular dynamics and evolution of proteins and other complexes.

## 1. Introduction

The ribosome is a large and universal RNA-protein complex that mediates protein synthesis. In recent decades, progress in imaging technologies fueled considerable advances in understanding its atomic structure. While first structures at a near-atomic resolution were established using X-Ray crystallography [1,2,3], the emergence of cryogenic electron microscopy (cryo-EM) has more recently led to a surge of new structures [4], encompassing multiple species, as well as various binding and conformational states. Being present in all life forms and different states, the ribosomal structure varies in its conformational and compositional aspects, both of which require quantitative tools to study. While conformational variations account for the different spatial configurations that a single ribosome can assume, differences in ribosome composition result from the diversification of structural components and their sequences. Besides the evolutionary differences in ribosomal composition across species, there has also been increasing evidence of ribosomal heterogeneity within individual cells and across tissues, suggesting some specialization of the ribosome for gene expression at the cellular and subcellular scales [5]. Conversely, this need to quantify these different forms of ribosomal heterogeneity across different scales illustrates certain limitations and computational challenges currently faced by structural biologists [6]. Standard algorithms for 3D reconstruction from cryo-EM image data [7,8] are restricted to classify structures in an arbitrary number of discrete states, and thus fail to capture the potential 3D motion underlying conformational heterogeneity [9]. Similarly, with individual 3D structures of ribosomes being confined to the current databases, computational tools are now needed to effectively compare the composition of available structures and quantify their variation.

The purpose of this review is to present the recent advances made in the quantification and extent of ribosome heterogeneity. In the first part, we will cover the multiple aspects and scales of heterogeneity, from the dynamical aspects of ribosomal motion, to differences of composition across species. As these various aspects of heterogeneity call for new methods and tools, we will focus in the second part on the computational challenges currently faced to exploit the information available from cryo-EM data. Overall, the development of these new computational methods for analyzing structural heterogeneity promises exciting new insights for the study of the ribosome and its biological implications.

## 2. The Multiple Sources of Heterogeneity in Ribosome Structures

**Figure 1:**
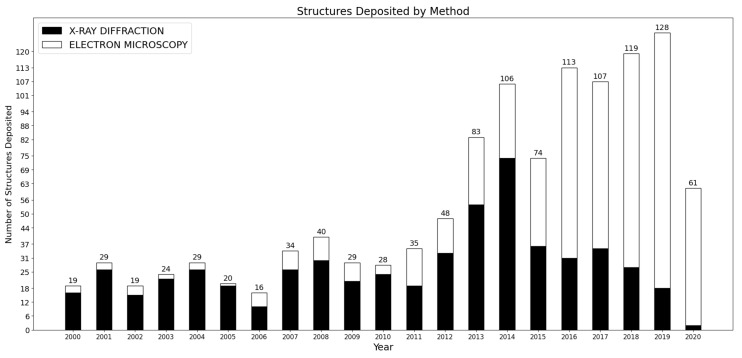
Number of structures related to the ribosomes and deposited in the Protein Data Bank (PDB) over the past 20 years. Data collected on rcsb.org [16].

Since the first structures described in 2000 that led to the Nobel Prize in Chemistry in 2009, the ribosome has been a central focus of structural biology, with more than 500 structures published since 2015 (see Figure 1). During this same period, cryo-EM also became primarily used to image the ribosome and its different parts, accounting for more than 80% of the structures deposited (compared with 38% from 2010 to 2015, and 18% in the decade of 2000–2010). This recent surge has allowed researchers to investigate various aspects of the ribosome heterogeneity. In the first part of this review, we shall describe these multiple sources of heterogeneity, considering time scales spanning from millions of years of evolution to micro-seconds underlying conformational changes. While recent studies of the evolution and functions of the ribosome are impossible to exhaustively summarize in this review, we want to emphasize here various recent works that use some in-depth exploration of the ribosome structure from cryo-EM, to investigate different forms of ribosome heterogeneity. Altogether, these studies also suggest that a more integrated approach can be useful to bridge the gap between evolutionary and functional studies, by understanding how the translational machinery displays the capacity to structurally evolve, to accommodate different environments and modulate its function.

### 2.1. Sequence and Structural Divergence across Species and Domains of Life

**Figure 2:**
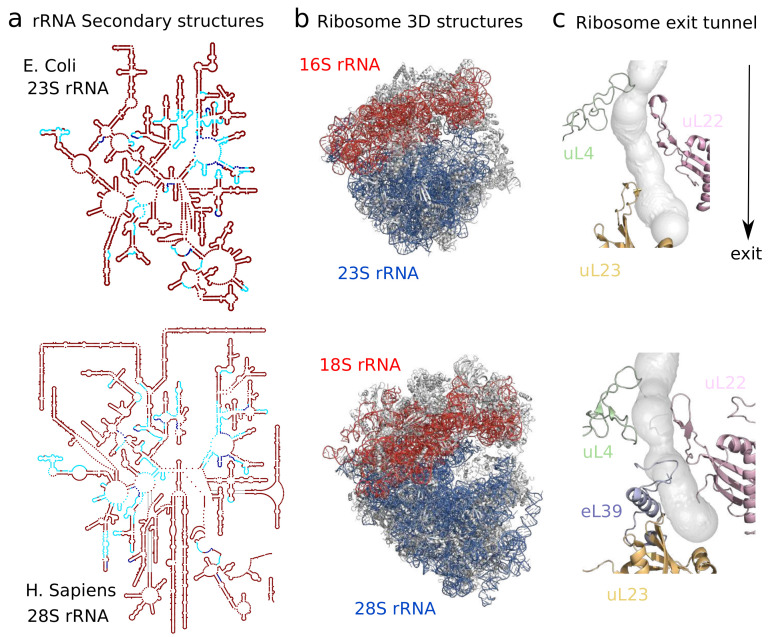
Comparison between the ribosome structures of *E. Coli* and *H. Sapiens* shows differences arising at different levels and scales. In contrast with prokaryotic 23S rRNA, constitutive of the large ribosomal subunit, eukaryotic 28S rRNA contains additional expansion segments inserted at specific positions in the common conserved rRNA core. Secondary structures are visualized in (**a**) using Ribovision [17], with conserved motifs in blue, following Doris et al. [18]. These differences, alongside variation in protein composition and sequence, affect the global 3D structure of the ribosome shown in (**b**). *E. Coli* and *H. Sapiens* cryoEM structures, visualized with Pymol, are taken from Fischer et al. [19] and Natchiar et al. [20]. The structural heterogeneity has a direct functional impact as shown in (**c**): At the ribosome exit tunnel through which the nascent polypeptide chain transits, the presence of eL39 at the exit or of an additional arm in uL4 which creates a second constriction site make the exit tunnel narrower and shorter in *H. Sapiens* [14].

As recent ribosome structures account for all domains of life and diverse families, they provide an important way to study heterogeneity across species. To illustrate this diversity, we report 20 different species for which ribosome cryo-EM structures have been recently published in Table 1, obtained by querying the Protein Data Bank (as done in Figure 1). Before the dominant usage of cryo-EM, earlier crystal structures already shed light on some main differences in size and composition between prokaryotic and eukaryotic ribosomes [10], with archaeal ribosomes sharing several components with eukaryotes that are absent in bacteria [11,12]. These differences are evolutionarily driven by the addition of eukaryotic rRNA expansion segments and modifications of ribosomal proteins, which can subsequently lead to important differences in specific regions of the ribosome, as shown in Figure 2a,b. A more specific example of where these differences are localized, illustrated in Figure 2 is the ribosome exit tunnel, a subcompartment of the ribosome that contains the nascent polypeptide chain [13]. We recently performed a more general comparative analysis of the exit tunnel [14] that indicates important geometric differences between eukaryotes and prokaryotes, especially at the constriction site region, where eukaryotic tunnels are more narrow than their prokaryotic counterparts. Interestingly, with the latest high quality maps reaching a resolution of 2 Å, detailed chemical interactions and specific chemical modifications of the ribosome can now be observed, leading to deeper phylogenetic analysis of ribosomal components and identification of structural conservation to the level of solvation [15].

Recent studies of the ribosome composition and structure for diverse species have contributed to draw a more intricate picture of the ribosome evolution. An important example of divergence among eukaryotes is the kinetoplastid family, which has been the object of several structural studies [21,22,23,24], showing ribosomes with fragmented rRNA’s that are comparable in size to prokaryotic counterparts, with nearly all the eukaryote-specific rRNA expansion segments missing. Similarly, during their evolution into organisms with highly compacted genomes, microsporidia have removed essentially all eukaryotic expansion segments and repurposed several ribosomal proteins to compensate for the extensive rRNA reduction [25]. On the prokaryotic side, bacteria with short genomes also commonly show a reduction of rRNA variation with loss of specific ribosomal proteins [26], suggesting some future work to visualize and confirm these changes through 3D structures. In addition, mitochondrial ribosomes present important morphological differences with cytoplasmic ribosomes [27]. As cryo-EM technology allows to computationally sort ribosomes of different classes from the image data, the past few years have seen various new structures of mitochondrial ribosomes from yeast, plants, mammals and other eukaryotic cells [27,28,29,30,31,32,33,34,35]. In contrast with bacteria, from which mitochondria originate according to the endosymbiotic hypothesis [36], new or modified ribosomal proteins in mitoribosomes form an extended network around the ribosomal RNA. This network can either be expanded or highly reduced [35], explaining how mitoribosomes dramatically diverge in composition and size (for more details, see the recent review by Tomal et al. [29]).

### 2.2. Consequences of Modifications at Single Sites

As sequence variability carries major differences in the ribosome structure across species, single mutations offer another source of structural heterogeneity within them. Without the need for crystallized structures, the structural and functional consequences of these modifications can be elucidated by cryo-EM. Although testing for every possible nucleotide mutation is a daunting task, focusing on key functional regions allows one to reasonably mitigate it. For example, a first standardized and complete mutational survey was recently produced for the Peptidyl Transferase Center (PTC) [37], a region located at the core of the ribosome and associated with peptide bond formation. Totaling 180 point mutations, this study indicates that despite the highly-conserved nature of the PTC, almost every nucleotide possesses certain mutational flexibility, so one or more mutations at these positions still permit full-length protein synthesis in vitro. To investigate the role of the ribosome in tumor and ribosomopathies [38,39], mutational surveys and genetic screenings have more generally identified specific sites and regions of the ribosome structure, which can serve as primary targets for drug treatment. Although there are still some limitations due to the low throughput of the technique and time to obtain structure at high resolution, using cryo-EM can explain how specific ligands can bind to ribosomes and inhibit their activity, offering a new perspective for structure-based drug design [40,41].

While this approach has been relatively recent in human [41,42,43,44], the determination of complex ribosomal structures with binding drugs has been an intense subject of study in bacteria. Superimposition and comparison with regular structures have explained how antibiotic drugs can target and modify specific sites of the ribosome, to interfere with different key steps during translation [45]. There is a variety of mechanisms for translation inhibition, as summarized in recent reviews [45,46], that involve both small and large subunits, including tRNA binding sites, the decoding center (also important for the formation of initiation complex), the polypeptide transferase center (PTC) and the exit tunnel. Conversely, mutations at these sites have been shown to potentially trigger antibiotic resistance [47,48,49]. Cryo-EM has been an important tool to investigate the causes of resistance coming from resistant mutant strains, as recently illustrated with the *S. aureus* erythromycin resistant mutant [50], or from species which diverge enough in structure like *Acinetobacter baumannii* [51], a Gram-negative plant pathogen that remarkably resists antibiotics through multiple mechanisms. In this regard, the development of new drugs and therapies that selectively target pathogens is essential and was the object of several other recent structural studies [52,53,54].

### 2.3. Heterogeneity within Cells and across Cell Types

The scope of ribosome heterogeneity also expands within cells and across tissues and cell types. Paralog or alternative ribosomal protein and rRNA genes provide a direct source of ribosome heterogeneity [55]. The extent to which this heterogeneity leads to some specialized function and regulation of gene expression is still elusive [5,56]. Yet, with the use of modern techniques in high-throughput sequencing and mass spectrometry, there has been over the past decade an accumulation of evidence supporting the existence of ribosomes with distinct protein composition and physiological function [57,58,59]. Under changes of conditions, development, or stress, the modulation of expression and stoichiometries of specific ribosomal proteins lead to “defects” that allow for specialization [57] but can also be the cause of disorders underlying ribosomopathies [60]. On the other hand, the mechanisms of repair and replacement of ribosomal proteins [61] can homogenize the ribosomal pool. These mechanisms also vary according to the cell type and spatial organization. For example, single cell comparative measurements of mRNA level between the soma and dendritic parts of neurons have surprisingly revealed higher abundance of some specific ribosome proteins in the dendritic region [62] (similar observation was also found in glial cells [63]). An interesting hypothesis, suggested by cell imaging, is that the ribosomal proteins in dendrites actually join pre-existing ribosomes, to maintain translation activity in axons [64], far from the nucleus where the ribosome is assembled.

In situ visualization of repaired or defective structures by cryo-EM would help to confirm this hypothesis but also reveals challenging, as one needs to generate enough samples for 3D reconstruction and classify the different particles according to these modifications. Cryo Electron Tomography (cryo-ET) provides an exciting direction for visualizing the ribosome in situ, e.g., interacting with organelle membranes or as parts of polysomes [65,66,67]. This dream of visualizing the molecular sociology of the cell [68] has spurred two major technological breakthroughs. On the experimental side, the development of focused ion beam milling techniques allow one to bypass the absorption problem with thick specimens [69], thus allowing access to native structures deep inside cells. On the data analysis side, the development of a unified framework for processing cryo-EM data has allowed researchers to break the traditional resolution barrier in cryo-ET and notably resolve the ribosome structure inside bacterial cells at 3.7 Å [70], paving the way for novel structural studies of the ribosome heterogeneity within cellular environments.

### 2.4. Conformational Heterogeneity and Molecular Motion

Translation involves major conformational changes of the ribosome, which gets assembled and translocates to the next codon at each elongation cycle. To capture these changes, as well as those of many other cotranslational processes, cryo-EM offers the ability to separate millions of sampled particles into multiple volume classes, which provide snapshots of the ribosome dynamics. From these different conformational states, one can infer a wide range of motions, such as multiple rotations relative to the LSU or the SSU [71], displacements at the intersubunit bridges [72], or more extreme flexibility of the stalks [73]. These motions are at play during the elongation cycle [73,74,75], initiation [76] and termination [77], as well as other cotranslational processes dictated by local interactions with various complexes, e.g., tRNA, elongation factor, translation inhibitors etc. [11,77,78]. On a related topic, it should also be noted that cryo-EM similarly led to considerable progress in elucidating the mechanisms of ribosome biogenesis (for more details, we refer to the recent reviews on the bacterial [79] and eukaryotic [80] ribosome assembly). While early studies of conformational heterogeneity using cryo-EM did not allow researchers to visualize intermediate states at a resolution less than 9 Å [81,82], ribosome structures characterizing different conformational changes can now be obtained at a higher resolution from 3 to 4 Å [77,83,84]. By sampling particles ∼10 ms or more after initiating a reaction, time-resolved cryo-EM [85] has recently helped to increase the number of intermediate conformations to include low-population structures (approximately 5 to 10 in the previously cited studies), with the latest study of elongating ribosome producing 33 states.

Despite offering an increasingly detailed view of the ribosome at different stages of translation processes, these multiple conformational states of the ribosome structure still offer a static overview of the conformational landscape. Yet, detailed 3D structures can serve as an important basis or complement for more direct studies of the underlying kinetics. Coarse-grained and atomistic molecular dynamics simulations take 3D structures as an input to model the thermodynamic and kinetic properties of the ribosome [86,87]. On the experimental side, understanding the 3D structure has been useful to guide and interpret single molecule fluorescence imaging experiments which offer time series data of the ribosome [78,88]. Beyond the standard approach in cryo-EM which leads to determine a finite set of 3D structures, a further challenge is to extract some more information on the conformational space that generates all the sampled images. In this regard, the ribosome is a reference model that is well studied and offers some important motion for testing new methods (which we shall cover in the next part). For example, the multibody refinement method in Relion, which allows one to mask some specific parts of the structure, is naturally suited to characterize the ribosome and its two subunits [89]. Other recent methods that infer how images lie in the continuous conformational space of a molecule [90,91] have also been recently proposed and showed good performance in resolving the ribosome main centers of motion.

## 3. Computational Challenges for Quantifying Heterogeneity from Cryo-EM Structures

Unraveling all of the aforementioned aspects of ribosome heterogeneity poses various computational challenges. This in turn has made the ribosome the center of modern developments in cryo-EM.

### 3.1. Data Integration for Structural Comparison

**Figure 3:**
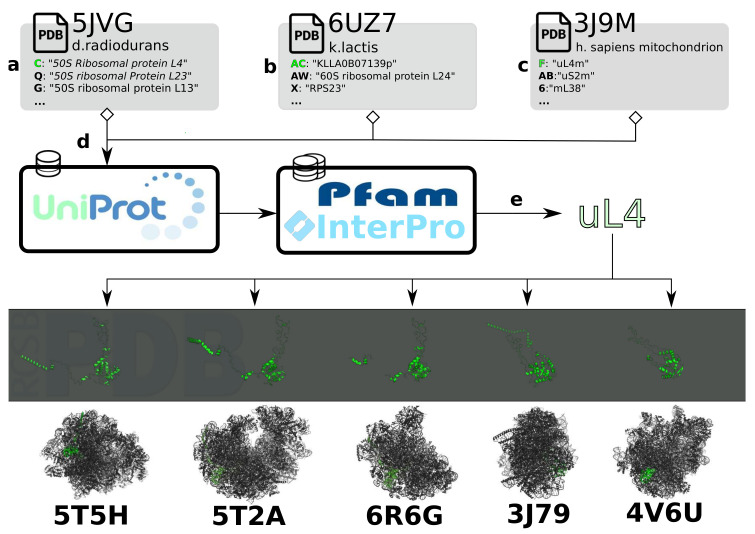
Processing of data and annotations for comparison of the ribosome across structures. (**a**–**c**) Subchain annotations in a *.mmcif/.pdb* file are heterogeneous and have to be converted to a single namespace to access homologous proteins across a diverse set of structures. (**d**) Protein classification databases provide information on families to which the subchains belong. (**e**) Sets of protein families are mapped to an identifier. In this case, the nomenclature proposed by Ban et al. [92] is used.

While the plethora of available structures makes a comparative analysis of ribosomal structures timely, performing such studies proves challenging in practice. First of all, in order to compare structures deposited by the community in a shared data bank [16], a common ontology is required for comparing proteins and the data associated with them across multiple pdb files (Figure 3a–c). One solution is to refer to Uniprot accession codes and/or InterPro families of the proteins (Figure 3d), but the naming of ribosomal proteins presents a specific obstacle for data integration. Due to historical contingency, many ribosomal proteins from different species were originally assigned the same name, despite being often unrelated in structure and function. To eliminate confusion, a nomenclature has been proposed to standardize known ribosomal protein names and provide a framework for novel ones [92]. While this nomenclature has been mostly adopted in recent structural studies, PFAM families and UniProt database as well as PDB still contain numerous references to earlier naming systems, as illustrated in Figure 3 for uL4. Given that members of certain PFAM super-families (*ex.PF01248, PF00467*) span multiple nomenclature classes, there remains a need for manual curation and disambiguation. By the same token, certain proteins belong to multiple PFAM families based on their sequence and some remain unclassified. These many-to-many mappings along with the differences in the classification methods employed by member-databases of InterPro make a fully-automated conversion mechanism between PFAM/InterPro and the proposed nomenclature (Figure 3e) problematic. Ambiguities of database searching in Uniprot have also been explicitly mentioned as an obstacle for the ribosome structure-based system to be adopted [93].

In light of these issues, there is a need for ribosome-centric databases that gather available 3D structures, associated protein data at multiple structural scales, and allow users to compare ribosome components across these structures. In addition, an important needed feature would be to provide enough flexibility to augment such databases with the publication of new structures. Previous efforts were made to build databases and interfaces for 3D alignment structures [94], or jointly visualize 1D, 2D and 3D structures of the ribosome [17]. Yet, they do not scale up to the recent increase of data and species available. Graph-databases [95] and GraphQL APIs are promising tools for this task, for their ability to accommodate and connect more heterogeneous data. Efforts within the structural bioinformatics community to adopt these technologies and increase connectivity are also notable [96] but are still far from being the go-to model.

### 3.2. Classification and Comparison of Ribosomal Components

**Figure 4:**
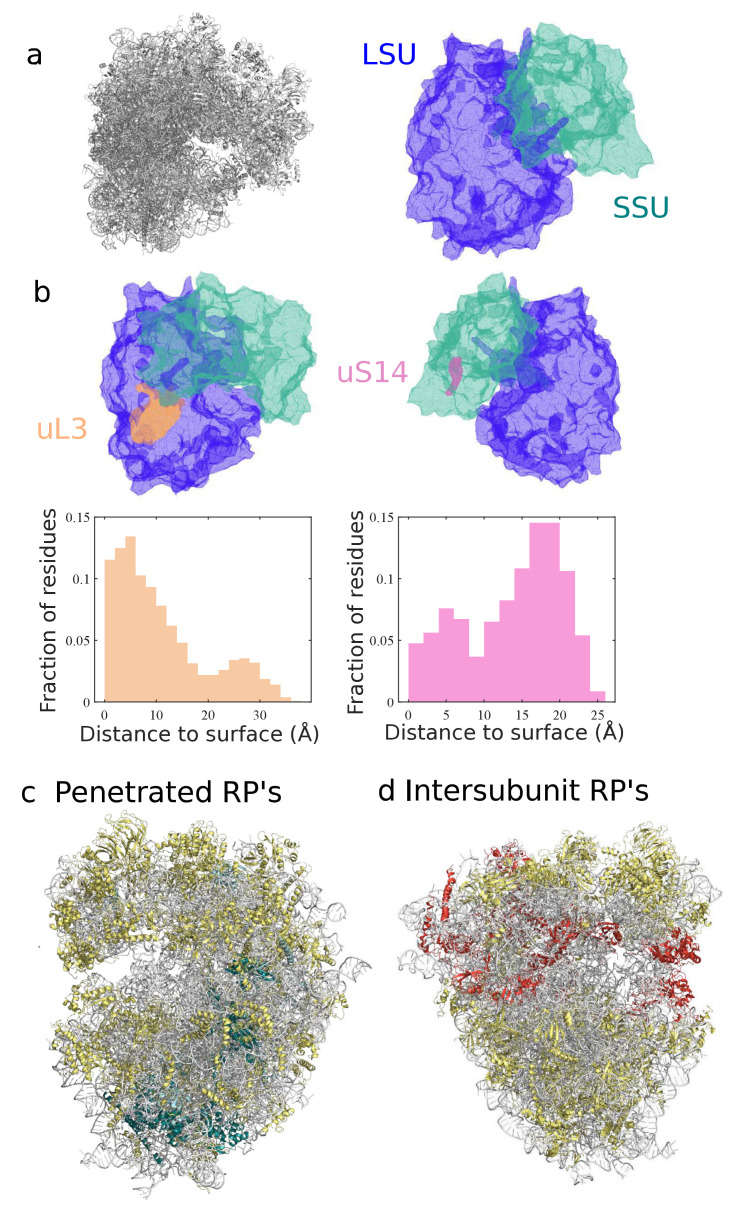
Interpolation of ribosome shape serves for protein spatial classification. Using a geometric descriptor called α-shape [101], we interpolated in (**a**) the surface of the human ribosome from the cryo-EM 3D structure [20]. Distance of any residue to the solvent-exposed surface can then be measured, yielding protein-specific distributions of distance, illustrated in (**b**) for two proteins of the large and small subunits, uL3 and uS14. This quantitative evaluation enables various spatial classifications of the ribosomal proteins, based on their degree of penetration inside the ribosome ((**c**), with deeply buried RP’s shown in blue) or localization in specific regions (as in (**d**), with intersubunit proteins shown in red).

A detailed comparative analysis of the ribosome structures can help to elucidate the extent and implications of the diversity of the ribosome and the various degrees of homology of ribosomal RNA and proteins [18,97]. Simple statistics, e.g., size or number of components are informative of major differences across domains of life. However, they do not fully take advantage of the spatial information provided by cryo-EM structures, or account for local variations. For example, although eukaryotic ribosomes are generally larger in size than bacterial ones, their exit tunnel is narrower with heterogeneous variations along it [14,98]. Various algorithms and computational methods adapted to molecular structures, based on tesselation [99,100,101] (illustrated in Figure 4a), or spectral geometry [102], can be used to encode the structure into geometric objects [103], and in particular compare ribosome geometric features. For example, by estimating the relative position of residues to the surface, one can separate proteins according to their degree of exposition to the solvent (see Figure 4b), which has been hypothesized as a key factor for differentiating proteins prone to ribosome repair [64] or with distinct electrostatic properties [104]. Overall, a more sound and quantitative approach can then help to develop standards to assess spatial properties such as solvent exposition, and various other properties of functional and evolutionary interests, e.g., the clustering or colocalization of proteins, such as intersubunit bridges, binding factors, and other key regions of the ribosome (see Figure 4c,d).

From an evolutionary perspective, the diversity of cryo-EM structures also allows one to treat the ribosome geometry at the molecular level as a quantitative trait, and thus establish direct association between conservation of structures and sequences. For such a complex and heterogeneous 3D object as the ribosome, it is yet challenging to find metrics that can properly detect evolutionary variations as done for sequence-based phylogenies. Our recent study of the geometry of the ribosome exit tunnel can be seen as a first attempt to do so [14]. Although the metric that we used, based on the radius variation along the tunnel [103], simplifies the geometry of the tunnel, it was still able to yield a robust hierarchical tree reflecting the species phylogeny. In addition, it allowed us to isolate the local regions explaining most of the geometric differences, revealing the presence of a second constriction site in the eukaryotic tunnel or reduced opening size at the exit port (see Figure 2c). Other biophysical properties, such as electric charges or hydrophobicity, have been shown to influence the translation dynamics [105,106,107]. More computational geometric tools and metrics should be developed in the future, to study other parts of the ribosome, compare more structures, and unravel the evolution and function of the ribosome.

### 3.3. Investigating Conformational Heterogeneity

**Figure 5:**
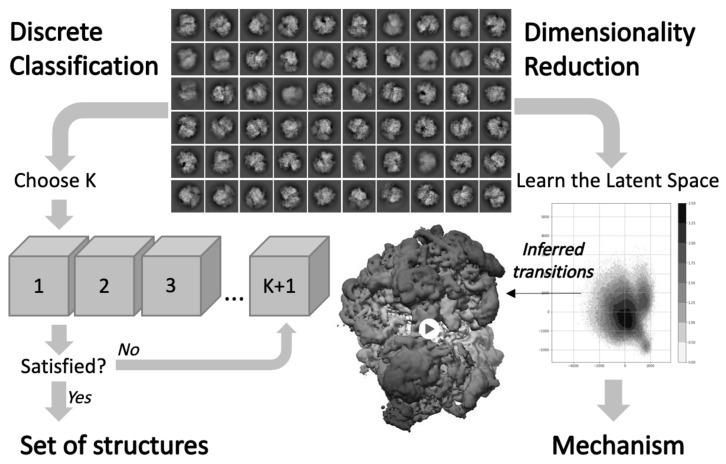
3D classification and inference of conformational heterogeneity from cryo-EM data. To study discrete heterogeneity (left), traditional 3D classification methods produce a finite number of structures. To study continuous heterogeneity (right), more recent approaches learn a dimensionally reduced representation of the conformational space, from which continuous transition pathways can be visualized to unravel biological mechanisms.

As highlighted in the first part of this review, the development of cryo-EM has led to determine multiple structures of the ribosome that reflect its conformational heterogeneity. The most popular computational method used to address conformational heterogeneity is referred to as 3D classification in most standard softwares used for single particle reconstruction [7,8,108] and actually corresponds to solving a mixture problem. In practice, the determination of multiple classes requires several rounds of classification using different initial references and different number of classes (see Figure 5), for which each image gets assigned [9,109]. The exact protocol varies from user to user and is more an art than a science. Without more systematic procedures and criteria to apply to the data, the evaluation and determination of an unknown number of states can unnecessarily mobilize time and computational resources. Methods for inferring the number of states have been investigated, notably by estimating the covariance matrix of the consensus 3D structure [110,111], but they are, to our knowledge, not implemented in standard software and costly to run with a large structure such as the ribosome.

Beyond discrete classification, the construction and inference of continuous motion is an important goal for improving our understanding of molecular behavior from cryo-EM data. Morphing-based techniques can be used to interpolate and visualize continuous trajectories between classes. In particular, our lab recently developed a morphing tool suited to perform transport-based interpolation between EM maps [112], while previous methods relied on mapping between two atomic models to avoid steric clashes [113]. From a theoretical point of view, this concept of continuous conformational heterogeneity follows the idea that the conformational space of the molecule is a finite dimensional manifold, also called latent space depending on the scientific community. The inference of continuous motion can be cast as an inverse problem aiming to reconstruct this manifold from the 2D images and the conformational landscape associated with the distribution of the cryo-EM images on this manifold (see Figure 5).

With unknown pose and microscope parameters, high signal-to-noise ratio, and limited sampling of particle images, the context of cryo-EM image formation makes this problem challenging. Yet, multiple approaches have been proposed to approximate the manifold of heterogeneous conformations. A few groups have proposed ways to approximate the manifold with a linear subspace, akin to principal component analysis [111,114], and a very similar method for 3D variability analysis was implemented in cryoSPARC [90]. The subsequent variability components are inferred, and for the ribosome, have been shown to capture shifiting and rotational subunit motions. Others have developed nonlinear methods to yield more accurate approximations. First, a method based on learning different manifold embeddings for clusters of images sharing similar viewing directions was developed and applied to model continuous deformations of the ribosome [115]. A more direct approach using all projection images regardless of their viewing direction was proposed to approximate the manifold of conformations [116], and it would be interesting to compare how well it performs on analyzing ribosome heterogeneity. Finally, cryoDRGN, a spatial variational auto encoder (VAE) architecture was developed to learn the latent space of conformational heterogeneity [91]. When applied on a ribosome dataset that had been previously carefully analyzed using a divide-and-conquer 3D classification approach, cryoDRGN showed the ability to directly map the relevant clusters on a low dimensional manifold that could then be further analyzed to understand how the different classes are topologically related. Despite their popularity, the use of neural networks for 3D reconstruction in cryo-EM is fairly new (see also [117,118]), suggesting promising directions for future research involving new learning architectures.

**Table 1:** Overview of species with ribosome cryo-EM structures solved at a resolution less than 3.8 Å. First column contains the species, with the domain they belong to (b: bacteria, a: archaea, e: eukarya).

Species/Organelles	Resolution	Reference	PDB Codename
*E. coli* (b)	2.2 Å (EM)	Stojkovic et al. (2020) [119]	6PJ6
*S. aureus* (b)	2.3 Å (EM)	Halfon et al. (2019) [50]	6S0Z
*T. cruzi* (e)	2.5 Å (EM)	Liu et al. (2016) [120]	5T5H
*S. cerevisea* (e)	2.6 Å (EM)	Tesina et al. (2020) [121]	6T4Q
*P. aeruginosa* (b)	2.8 Å (EM)	Halfon et al. (2019) [122]	6SPB
*H. sapiens* (e)	2.9 Å (EM)	Natchiar et al. (2017) [20]	6EK0
*A. baumanii* (b)	2.9 Å (EM)	Morgan et al. (2020) [51]	6V3D
*L. donovani* (e)	2.9 Å (EM)	Zhang et al. (2016) [24]	5T2A
*Mitochondria* (*H. Sapiens*)	3.1 Å (EM)	Amunts et al. (2015) [33]	3J9M
*M. smegmatis* (b)	3.2 Å (EM)	Hentschel et al. (2017) [123]	5O60
*P. falciparum* (e)	3.2 Å (EM)	Wong et al. (2014) [124]	3J79
*T. gondii* (e)	3.2 Å (EM)	Li et al. (2017) [21]	5XXB
*T. b. brucei* (e)	3.3 Å (EM)	Saurer et al. (2019) [125]	6SGB
*M. tuberculosis* (b)	3.4 Å (EM)	Yang et al. (2017) [126]	5V7Q
*T. vaginalis* (e)	3.4 Å (EM)	Li et al. (2017) [21]	5XY3
*C. thermophilum* (e)	3.5 Å (EM)	Cheng et al. (2019) [127]	6RXU
*K. lactis* (e)	3.6 Å (EM)	Huang et al. (2020) [128]	6UZ7
*O. cuniculus* (e)	3.7 Å (EM)	Shanmuganathan et al. (2019) [129]	6R6G
*Chloroplast* (*Spinacia*)	3.8 Å (EM)	Ahmed et al. (2017) [130]	5X8T
*B. subtilis* (b)	3.8 Å (EM)	Beckert et al. (2017) [131]	5NJT
